# The use of host cell DNA methylation analysis in the detection and management of women with advanced cervical intraepithelial neoplasia: a review

**DOI:** 10.1111/1471-0528.16395

**Published:** 2020-08-09

**Authors:** WW Kremer, RDM Steenbergen, DAM Heideman, GG Kenter, CJLM Meijer

**Affiliations:** ^1^ Department of Pathology Cancer Centre Amsterdam Amsterdam UMC Vrije Universiteit Amsterdam Amsterdam The Netherlands; ^2^ Obstetrics and Gynaecology Cancer Centre Amsterdam Amsterdam UMC Vrije Universiteit Amsterdam Amsterdam The Netherlands; ^3^ Centre for Gynaecological Oncology Amsterdam Antoni van Leeuwenhoek‐Netherlands Cancer Institute and Amsterdam UMC Amsterdam The Netherlands

**Keywords:** cervical cancer screening, cervical intraepithelial neoplasia, DNA methylation, human papillomavirus

## Abstract

This paper briefly reviews the role of hypermethylation of host cell genes in cervical carcinogenesis and discusses potential clinical applications of methylation analysis in the management of high‐risk HPV (hrHPV) ‐positive women. We argue that methylation assays can be used: 1. for primary triage of hrHPV‐positive women to detect cervical cancer and advanced cervical intraepithelial neoplasia (CIN); 2. as secondary triage for women with minor cytological abnormalities to identify those with the highest risk of CIN3 or worse; 3. as exit test for women leaving the screening programme to identify cervical cancer and advanced CIN; and 4. to support management of CIN.

**Tweetable abstract:**

This paper discusses potential clinical applications of DNA methylation analysis in the management of women with a high‐risk HPV infection.

## Tweetable abstract

This paper discusses potential clinical applications of DNA methylation analysis in the management of women with a high‐risk HPV infection.

## Introduction

Persistent infection with a high‐risk type of human papillomavirus (hrHPV) is a necessary cause for cervical cancer.^1^ The development of invasive cervical cancer through premalignant lesions (cervical intraepithelial neoplasia [CIN] graded 1–3) is a slow process that can take up to 30 years from initial hrHPV infection, allowing detection and treatment of early‐stage disease.^2^ Primary hrHPV DNA testing is currently preferred for cervical screening over cytology because of its superior sensitivity and high negative predictive value for high‐grade CIN or worse (CIN2+).^3^ However, most hrHPV infections are productive infections, i.e. producing new virions, and do not give rise to cervical cancer, leading to a 3–5% lower specificity of the HPV test compared with cytology.^4^ Therefore, a triage test is required to distinguish hrHPV‐positive women with clinically relevant cervical lesions from those with transient infections. New biomarkers, such as DNA methylation of specific host cell genes, are being evaluated as alternative triage methods to further improve risk stratification of hrHPV‐positive women.

Here we will briefly highlight the role of DNA methylation in cervical carcinogenesis and discuss the potential clinical applications of DNA methylation analysis in the detection and management of women with cervical neoplastic lesions. We will focus on methylation markers that have been clinically validated for the detection of cervical cancer, CIN3 and CIN2 in cervical scrapes and self‐collected cervico‐vaginal cells, and that are currently available within commercial or research methylation assays.

## DNA methylation and cervical carcinogenesis

Cervical carcinogenesis is driven by the viral oncoproteins, E6 and E7. Their transcription and the HPV productive life cycle in differentiated epithelium relies on interactions with multiple cellular proteins and complex epigenetic remodelling of the viral chromatin, including both histone modifications and DNA methylation.^5^ This epigenetic programme is disrupted during malignant transformation. The interaction of E6 and E7 with various epigenetic regulators, such as DNA methyltransferases, histone‐modifying enzymes and subunits of chromatin remodelling complexes, affect the transcription of host cell genes.^6^

**Figure 1:**
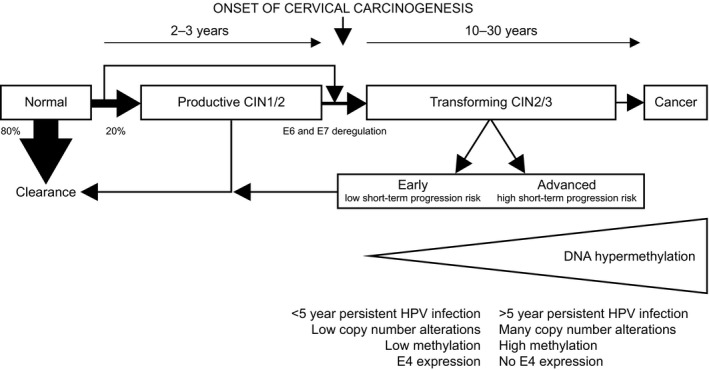
Schematic representation of the various outcomes of hrHPV infection of cervical epithelial cells and characteristics of early versus advanced transforming lesions. Most hrHPV infections are cleared by an effective immune response without causing cellular abnormalities (transient infections). Productive infections can give rise to productive cervical intraepithelial neoplasia (CIN, mainly CIN1 and a subset of CIN2), of which the majority regresses spontaneously within 1 or 2 years. Transforming infections are characterised by deregulated E6 and E7 expression and are associated with transforming CIN (the remaining subset of CIN2 and CIN3). Transforming CIN is a heterogeneous disease with variable duration of lesion existence and includes both progressive and regressive lesions. On routine haematoxylin & eosin‐stained sections these lesions cannot be distinguished. Current data support the division of transforming CIN lesions into early transforming lesions, characterised by a <5 year preceding HPV infection, few copy number alterations, low methylation levels and E4 expression and advanced transforming lesions, characterised by a ≥5 year preceding HPV infection, cancer‐like copy number alterations, high methylation levels and no E4 expression. As increases in genetic and epigenetic alterations are associated with disease progression, early transforming lesions are thought to have a low short‐term progression risk to cervical cancer, whereas advanced transforming lesions are thought to have a high short‐term progression risk.^8^, ^47^, ^50^

The best‐studied host cell modification in cervical cancer is DNA methylation, which involves the covalent binding of a methyl group to the 5′ position of a cytosine molecule in CpG dinucleotides. Besides global hypomethylation, the overall loss of methylation during carcinogenesis, resulting in chromosomal instability,^7^ and the silencing of tumour suppressor genes by local hypermethylation of CpG‐rich promoter regions contribute to cancer development.^6^, ^8^ In vitro models have shown that methylation of (candidate) tumour suppressor genes occurs at the stage of immortalisation and increases with progression to a tumorigenic phenotype.^9^, ^10^ This progressive increase in DNA methylation seen in vitro is comparable to the observations in CIN and cervical cancer tissues, also demonstrating a gradual increase in methylation with progression to cancer (see Figure 1).^11^ The functional relevance of methylation‐mediated gene silencing during HPV‐induced carcinogenesis has been demonstrated for a subset of currently known methylation targets, including *miR124‐2*, *CADM1*, *MAL* and *PAX1*.^12^, ^13^, ^14^, ^15^, ^16^, ^17^, ^18^, ^19^

### Detection of DNA methylation

Detection of methylated host cell DNA can be performed by different molecular methods and on different sample types including cervical tissue, cervical scrapes, self‐collected cervico‐vaginal cells and even urine.^8^, ^20^ Most methods require a bisulphite conversion step, a chemical reaction of sodium bisulphite with DNA that converts unmethylated cytosines to uracil, whereas methylated cytosines remain unaffected. Quantitative methylation‐specific PCR is most commonly used to detect methylation of targeted sequences, as it requires a minimal amount of input DNA, is suitable for high‐throughput settings and is highly reproducible. Another sensitive method used for clinical application is pyrosequencing, a method providing an absolute level of methylation.^21^

### Available methylation assays

Although many methylation targets have been identified using both targeted and genome‐wide approaches, extensive validation for use in clinical practice and development into a commercial or research methylation assay has only been performed for some (Table 1). These include the marker panels *CADM1* and *MAL*, later supplemented with *miR124‐2* (PreCursor‐M^®^, Self‐Screen B.V., Amsterdam, the Netherlands), *FAM19A4* and *miR124‐2* (QIAsure Methylation Test®, Qiagen, Hilden, Germany), *ASTN1*, *DLX1*, *ITG4*, *RXFP3*, *SOX17* and *ZNF671* (GynTect^®^, Oncgnostics, Jena, Germany), *EPB41L3* combined with viral target from four HPV types (HPV16L1, HPV16L2, HPV18L2, HPV31L1 and HPV33L2; S5 Classifier, research assay), *POU4F3* (Confidence Marker™, Neumann Diagnostics, Budapest, Hungary) and *PAX1* (Cervi‐M^®^, Ingenuity Healthcare, Mumbai, India). Other promising target genes include *JAM3*, *C13ORF18*, *TERT*, *ANKRD18CP*, *CDH6*, *GFRA1*, *LHX8*, *PCDHA4*, *PCDHA13*, *SOX1* and *ZNF582*.^11^, ^21^, ^22^, ^23^, ^24^

**Table 1:** Overview of commercial or research host cell DNA methylation assays and their performance for the detection of CIN3 or worse in cervical scrapes or self‐collected specimens in different clinical settings

Marker (panel)	Assay	Reference	HPV	Sample type	*n*	CIN3 (%)	ICC (%)	Sens.	Spec.	NPV	PPV	Comment	
**Screening populations**													
*FAM19A4/miR124‐2*	QMSP	Bonde, subm.	HPV+	Cervical scrapes	2384	228 (9.6%)	20 (0.8%)	77.2	78.3	96.9	28.3	End point CIN3; European screening populations	
*FAM19A4/miR124‐2*	QMSP	[70]	HPV+	Cervical scrapes	979	109 (11.1%)	6 (0.6%)	71.3	78.3	94.8	33.0	Cross‐sectional data	
								—	—	87.2	—	14‐year NPV	
*FAM19A4/miR124‐2*	QMSP	[69]	HPV+	Cervical scrapes	1025	192 (18.7%)	19 (1.9%)	—	—	83.7	60.2	14‐year NPV and PPV	
*CADM1/MAL*	QMSP	[63]	HPV+	Cervical scrapes	234	38 (16.2%)	3 (1.3%)	68.4	75.5	92.5	35.1	Consecutive series from screening cohort	
*EPB41L3*/HPV16L1/HPV16L2/HPV18L2/HPV31L1/HPV33L2*	PSQ	[34]	HPV+	Cervical scrapes	257	44 (17.1%)	8 (3.1%)	93.2	41.8	—	24.8	Case–control within screening cohort; end point CIN3	
*EPB41L3*/HPV16L1/HPV16L2/HPV18L2/HPV31L1/HPV33L2*	PSQ	[65]	HPV+	Cervical scrapes	341	18 (5.3%)	1 (0.3%)	84	63	—	—	Selection of women from screening cohort; CIN2 (*n* = 20) lesions were excluded from analyses	
**Non‐attendee population**													
*FAM19A4/miR124‐2*	QMSP	[28]	HPV+	Self‐samples (brushes)	254	68 (26.8%)	4 (1.6%)	69.4	76.4	86.3	53.8	Performance data from validation set; brush‐ and lavage‐collected samples from (mostly) same patients	
				Self‐samples (lavage)	389	72 (18.5%)	6 (1.5%)	70.5	67.8	90.2	35.5		
*CADM1/MAL*	QMSP	[33]	HPV+	Cervical scrapes	364	56 (15.4%)	6 (1.6%)	69.4	71.2	91.9	33.1		
**Gynaecological referral populations/Selection of archived material**													
*FAM19A4/miR124‐2*	QMSP	[30]	HPV+/−	Cervical scrapes	538	66 (12.3%)	6 (1.1%)	77.8**	69.3**	98.3^**^	36.4^**^	Women with abnormal cytology referred for colposcopy	
*CADM1/MAL/miR124‐2*	QMSP	[27]	HPV+	Cervical scrapes	247	16 (6.5%)	79 (32.0%)	94.7	78.9	—	—	Selection of archived material	
*EPB41L3*/HPV16L1/HPV16L2/HPV18L2/HPV31L1/HPV33L2**	PSQ	[68]	HPV+	Cervical scrapes	1493	373 (25.0%)	—	90	49	—	51	Women with abnormal cytology referred for colposcopy; end point CIN2/3 (*n* = 556)	
*ASTN1/DLX1/ITGA4/RXFP3/SOX17/ZNF671*	QMSP	[37]	HPV+/−	Cervical scrapes	280	50 (17.9%)	5 (1.8%)	64.8**	94.6**	91.7**	74.5**	Residual samples from routine screening (NILM cytology) and from patients with CIN1+ on histology	
*ASTN1/DLX1/ITGA4/RXFP3/SOX17/ZNF671*	QMSP	[39]	HPV+/−	Cervical scrapes	306	88 (28.8%)	5 (1.6%)	67.7**	82.6**	—	—	Women referred for colposcopy	
			HPV+/−					64.5	88.7	—	—		
*POU4F3*	QMSP	[40]	HPV+	Cervical scrapes	1287	65 (5.1%)	12 (0.9%)	89.6	60.9	—	—	Mixed screening population and women referred for cone biopsy or hysterectomy; ICC cases included AIS and CIS	
*PAX1*	QMSP	[44]	HPV+/−	Cervical scrapes	449	87 (19.4%)	71 (15.8%)	69.6**	81.8**	—	—		
*PAX1*	QMSP	[42]	HPV+/−	Cervical scrapes	346	42 (12.1%)	30 (8.7%)	64**	91**	—	—	Women with low‐ or high‐grade cytology referred for colposcopy and controls from routine screening (NILM cytology)	
			HPV+/−					57	96	—	—		
*PAX1*	QMSP	[43]	HPV+/−	Cervical scrapes	419	32 (7.6%)	4 (1.0%)	86**	85**	—	—	Mixed screening population and women referred with abnormal cytology	
*PAX1*	QMSP	[45]	HPV+/−	Cervical scrapes	321	24 (7.5%)	1 (0.3%)	52.0**	90.3**	—	—	Women with ASC‐US cytology referred for colposcopy	

AIS, adenocarcinoma in situ; ASC‐US, atypical squamous cells of unknown significance; CIN, cervical carcinoma in situ; CIS, cervical intraepithelial neoplasia, graded 1–3; HPV, human papillomavirus; ICC, invasive cervical cancer; *n*, samples size; NILM, negative for intraepithelial lesion or malignancy; NPV, negative predictive value; PPV, positive predictive value; PSQ, pyrosequencing; QMSP, quantitative methylation‐specific polymerase chain reaction; Sens., sensitivity; Spec., specificity.

* Combination of cellular and viral targets.

** Performance evaluated independent of HPV result.

### Methylation in cervical cancer samples

Published data consistently show high methylation levels of most markers in nearly all cervical cancer specimens (tissue, scrapes or self‐samples), resulting in high accuracy for cervical cancer. A recent study evaluating a world‐wide series of over 500 cases of cervical cancer, including rare histotypes and HPV‐negative carcinomas, showed that 98.3% tested positive for *FAM19A4/miR124‐2* methylation.^25^ Methylation positivity was independent of histotype, International Federation of Obstetrics and Gynecology stage, HPV status and genotype, sample type and geographical region. This high cross‐sectional sensitivity for cervical cancer was also shown among hrHPV‐positive women from a Dutch cervical screening cohort.^26^ Smaller series of cervical carcinomas were also evaluated for *FAM19A4* alone, *EPB41L3* (with and without viral markers), *CADM1/MAL/miR124‐2*, *ASTN1/DLX1/ITGA4/RXFP3/SOX17/ZNF671* and *POU4F3,* all showing detection rates well over 90%.^27^, ^28^, ^29^, ^30^, ^31^, ^32^, ^33^, ^34^, ^35^, ^36^, ^37^, ^38^, ^39^, ^40^ For *PAX1*, positivity rates of at least 80% have been reported.^41^, ^42^, ^43^, ^44^, ^45^, ^46^

### Methylation in CIN lesions

Host cell DNA methylation analysis of cervical samples is a promising tool to identify hrHPV‐positive women with clinically relevant cervical lesions. Methylation levels of promoter regions of specific host cell genes increase with increasing CIN grade and are extremely high in cervical cancer. As genetic and epigenetic alterations necessary for cervical cancer progression accumulate over time, high methylation levels in these regions are likely to be associated with advanced cervical lesions with a longer duration of lesion existence.^8^ In support of this hypothesis is the finding that CIN2/3 lesions associated with a long‐term hrHPV infection (≥5 years), used as a proxy for duration of lesion existence, and a DNA copy number profile similar to cervical cancer had significantly higher methylation levels compared with CIN2/3 lesions associated with a short‐term hrHPV infection (<5 years) and few to no copy number alterations.^47^, ^48^, ^49^ Additional arguments were provided by van Zummeren et al., who showed that CIN lesions expressing the HPV E4 protein, indicating a productive HPV infection, were associated with low methylation levels, and Louvanto et al., who showed that high methylation levels are predictive of progression of untreated CIN2 lesions in women up to age 30 years.^50^, ^51^ Taken together, these available data argue that methylation assays detect advanced CIN lesions in need of treatment.

## Use of methylation analysis in cervical screening

### Primary triage of hrHPV‐positive women

Cervical screening programmes based on primary hrHPV testing require triage of hrHPV‐positive women, but consensus on the optimal triage test is currently lacking. Triage strategies require a good balance between safety, i.e. high sensitivity for cervical cancer and CIN3 (CIN3+), and screening‐related burden, i.e. minimal over‐referral of women without clinically relevant cervical lesions. Although the optimal balance varies between regions depending on locally accepted risk thresholds, available resources and population characteristics, the following aspects are generally required for triage tests within cervical screening programmes: 1. high reproducibility; 2. high cross‐sectional sensitivity for CIN3+, leading to a high negative predictive value (~98%); 3. high specificity, leading to high positive predictive value of at least 20% (the Netherlands) or 5–10% (USA) and 4. long‐term safety: longitudinal data evaluating the long‐term CIN3+ risk of triage‐negative women are required to determine appropriate screening intervals with a chosen triage strategy.^52^

Methods for triage of hrHPV‐positive women as used in current cervical screening programmes include cytology, HPV16/18 genotyping and repeat hrHPV testing. Although these triage strategies have acceptable clinical performance, there are some important drawbacks. Cytology triage cannot be performed on self‐collected specimens and its use is limited by its subjective nature, leading to suboptimal sensitivity and the need for repeat testing. Additional staining of cytology slides for p16^INK4a^ and ki‐67 has been shown to mostly improve the specificity for CIN3+.^53^, ^54^ However, as with sole cytology, interpretation remains observer‐dependent and validation of this triage strategy is still ongoing. Triage by HPV16/18 genotyping or repeat hrHPV testing has the benefit of objective test results, but both methods have to be combined with cytology to have sufficient clinical performance.^55^ A good alternative method for triage of hrHPV‐positive women is methylation analysis, with major benefits including high reproducibility, objectivity and applicability on both clinician‐collected and self‐collected cervical specimens.^28^, ^56^ These characteristics can facilitate full molecular screening with highly reproducible results.

#### Cross‐sectional performance of methylation markers for primary triage of hrHPV‐positive women

The cross‐sectional clinical performance of different methylation markers for the detection of CIN3+ has been studied using samples from different settings: cervical screening, screening non‐attendees and gynaecological referral populations (Table 1). Data from individual markers included in these assays are shown in the supplementary material (Table S1). As negative and positive predictive values for CIN3+ are influenced by the composition of the population tested, we will focus on the sensitivity and specificity of the different markers. CIN2 is not taken as an end point because it reflects a heterogeneous group of lesions and the diagnosis is moderately reproducible.^57^, ^58^ It should be noted that only marker panel *FAM19A4/miR124‐2* has been evaluated in large, population‐based cervical screening populations, and that most studies used either smaller, selected series from screening populations, or samples from gynaecological referral populations (indicated in Table 1 and the Supplementary material, Table S1).

The performance of *FAM19A4* and/or *miR124‐2* methylation has been studied extensively, showing consistently good sensitivity and specificity for the detection of CIN3+ in both clinician‐collected cervical scrapes (sensitivity 68.2–86.7%, specificity 60.6–79.3%) and self‐collected specimens (sensitivity 65.3–70.5%, specificity 67.8–81.3%).^28^, ^29^, ^49^, ^59^, ^60^, ^61^, ^62^ Methylation analysis of *CADM1* and/or *MAL*, and later with the addition of *miR124‐2*, has shown good performance for the detection of CIN3+ in cervical scrapes (sensitivity 68.0–94.7%, specificity 50.7–78.9%) and self‐collected specimens (sensitivity 64.9–71.6%, specificity 55.3–70.0%).^27^, ^33^, ^61^, ^63^, ^64^ Good sensitivity has also been described for *EPB41L3*, particularly when combined with methylation analysis of defined HPV genes of the L1 and L2 region. Depending on the setting and the inclusion of viral methylation markers, CIN3 or CIN3+ sensitivities ranging from 67.0 to 93.2% and specificities ranging from 41.8 to 85.0% have been reported.^34^, ^35^, ^36^, ^65^, ^66^, ^67^, ^68^ In a small series of self‐collected samples from a non‐attendee population, *EPB41L3* showed good sensitivity and specificity for CIN3+.^36^ The five‐gene marker panel consisting of *DLX1*, *ITGA4*, *RXFP3*, *SOX17* and *ZNF671*, later adjusted with the addition of a sixth marker *ASTN1*, has been evaluated for the detection of CIN3+ among women visiting gynaecological outpatient departments (with or without hrHPV testing), showing sensitivities and specificities ranging from 64.5 to 76.2% and 76.0 to 94.6%, respectively.^37^, ^38^, ^39^ *POU4F3* has been evaluated in a mixed screening and referral population for triage of hrHPV‐positive women, showing CIN3+ sensitivity of 89.6% and specificity of 60.9%.^40^ Although *PAX1* has been evaluated in several Asian referral populations, only one study reported its performance as a triage marker for the detection of CIN3+ (sensitivity 57%, specificity of 96%), whereas the others reported the performance of the marker without previous hrHPV testing (sensitivity 44.1–86.0%, specificity 81.8–96.0%).^41^, ^42^, ^43^, ^44^, ^45^, ^46^

#### Longitudinal performance of methylation markers for primary triage of hrHPV‐positive women

Longitudinal CIN3+ risk data after a negative triage test are needed to determine the safety of the chosen screening interval. Longitudinal data on the performance of methylation triage of hrHPV‐positive women is only available for the marker panel *FAM19A4/miR124‐2*. Long‐term follow‐up data from a Dutch cervical screening cohort showed that the cervical cancer risk among hrHPV‐positive women after 14 years is lower following a negative *FAM19A4/miR124‐2* methylation result compared with a negative cytology result and that the CIN3+ risk is similar following a negative methylation or cytology result.^26^, ^69^ This lower long‐term CIN3+ risk after a negative *FAM19A4/miR124‐2* methylation test was confirmed in another study from the Netherlands.^70^

### Secondary triage for CIN3 or worse of women with ASC‐US/LSIL cytology

Current HPV‐based cervical screening programmes often use cytology for triage of hrHPV‐positive women, referring all women with abnormal cytology (≥atypical squamous cells of unknown significance [ASC‐US]) at baseline or after 6 months for colposcopy. Although this triage strategy significantly improves the specificity of the primary hrHPV test, over‐referral of women without clinically relevant lesions remains an issue, particularly among young women with low‐grade cytological abnormalities (ASC‐US or low‐grade squamous intraepithelial lesion [LSIL]). The latest monitoring report from the Dutch HPV‐based national screening programme illustrates this issue of over‐referral: 21% of hrHPV‐positive women (aged ≥30 years) had ASC‐US or LSIL cytology at baseline and at least 48% of all hrHPV‐positive women did not have clinically relevant disease (CIN1 or less).^71^

Recent data from the VALID‐screen study, a multicentre study for the validation of *FAM19A4/miR124‐2* methylation in European countries, indicate that the referral rate could be lowered by approximately 34% by secondary triage of hrHPV‐positive women with ASC‐US/LSIL cytology using *FAM19A4/miR124‐2* methylation, while still detecting all cervical carcinomas and at least 70% of CIN3 lesions (Bonde et al., submitted). These data suggest that *FAM19A4/miR124‐2* methylation analysis could be used to distinguish among women with ASC‐US/LSIL cytology those who require immediate colposcopy referral versus re‐testing for hrHPV and methylation within 3 years follow up in HPV‐based as well as in cytology‐based screening programmes. Similar results have been published for *PAX1*, although without prior hrHPV testing, detecting at least 50% of CIN3 and all cervical carcinomas among women with minor cytological abnormalities.^43^, ^45^

### Exit test for women leaving the screening programme

A significant proportion of incident cervical cancers occur in elderly women outside the screening age. Because of the decreased balance between efficiency and harms, most countries screen women up to age 60 or 65 years. In this age‐group, the relatively low specificity of the hrHPV test is less problematic because of the lower HPV prevalence and fewer consequences of overtreatment. At the same time, some cervical cancers may be missed by hrHPV testing or by cytology triage testing, because of a higher frequency of hrHPV‐negative lesions and lower sensitivity of cytology testing at an older age.^72^, ^73^ With the high sensitivity of methylation analysis for cervical carcinomas, it would be an interesting option to combine hrHPV testing with methylation analysis at the final screening round, referring all women who test positive for either test for gynaecological examination (co‐testing). Vink et al.^25^ showed that 90% of hrHPV‐negative cervical carcinomas were positive for *FAM19A4/miR124‐2* methylation, suggesting that such a strategy may lead to earlier detection of women with cervical cancer and advanced CIN lesions.

### Management of hrHPV‐positive women of childbearing age

Women with a positive cervical screening result, whether performed within a population‐based screening programme or because of specific complaints, should be referred for colposcopy. Clinical management of these women is based on the CIN grade of a colposcopy‐directed biopsy. All CIN3 and most CIN2 lesions are treated surgically, whereas CIN1 lesions are generally managed with a watch‐and‐wait policy. Conservative management can also be considered in young women with CIN2, as spontaneous regression rates are high: up to 60% of CIN2 and 32% of CIN3 lesions regress spontaneously.^74^, ^75^, ^76^ Approximately 5% of untreated CIN2 and 12–31% of untreated CIN3 lesions ultimately progress to cancer, depending on the duration of lesion existence.^2^, ^74^, ^76^ Current histopathological assessment of cervical biopsies cannot distinguish lesions with a low risk of progression to cervical cancer from those with a high progression risk. This leads to overtreatment and associated morbidity, most notably an increased risk of adverse pregnancy outcomes in subsequent pregnancies.^77^, ^78^, ^79^ As most women with CIN2 and CIN3 lesions are of reproductive age, biomarkers to improve risk stratification and prevent overtreatment are needed. Several potential biomarkers have been evaluated, such as HPV genotyping, HPV viral load and immunohistochemical staining of cervical biopsies by p16^ink4a^ and Ki‐67, but none of these markers has been shown to have clinical prognostic value or to be able to predict progression at the individual patient level.^80^ Methylation analysis has emerged as a promising prognostic marker because of its specific sensitivity for advanced CIN lesions and cervical cancer.

The first data from a prospective longitudinal study on the value of methylation analysis in predicting the outcome of CIN lesions were recently published by Louvanto et al.^52^ In this study, women under the age of 30 years and diagnosed with a CIN2 lesion were managed conservatively with 6‐monthly follow‐up visits up to 2 years. High methylation of host cell marker *EPB41L3* combined with viral methylation markers (S5 classifier) were associated with progression to CIN3. A similar study is ongoing in the Netherlands, evaluating the value of *FAM19A4/miR124‐2* methylation in the prediction of regression or non‐regression in untreated women.^81^ This study also includes women with CIN3 and women aged up to 55 years. If their prognostic value is confirmed, methylation assays could be used to guide clinical management of women diagnosed with a CIN lesion.

### Use of methylation analysis for cervical screening in women living with HIV

The disease burden of both cervical cancer and HIV is disproportionally high in low‐ and middle‐income countries: >85% of global cervical cancer cases and >95% of all HIV infections occur in these regions.^82^, ^83^ Mainly because of HIV‐related immunodeficiency, women living with HIV are at increased risk for HPV infection, CIN lesions and cervical cancer.^84^, ^85^, ^86^ Cervical screening is extremely relevant in women living with HIV, but because of poverty, lack of resources and infrastructure, and limited access to health care, implementation of organised screening programmes in low‐ and middle‐income countries has proven to be challenging.^87^ Full molecular screening using methylation analysis could be used to improve cervical screening in low‐ and middle‐income countries, and women living with HIV in particular.

Studies on host cell methylation analysis in cervical scrapes of women living with HIV show promising results. The clinical performance of host cell methylation analysis for the detection of CIN3+ in these women has only been evaluated for some markers, including *FAM19A4/miR124‐2*, *EPB41L3* and *CADM1/MAL/miR124‐2*.^88^, ^89^, ^90^, ^91^ Similar to HIV‐uninfected women, methylation levels increase with cervical disease severity and are extremely high in cervical cancer in women living with HIV.^88^, ^89^, ^90^, ^92^ These data suggest that also in women living with HIV, high methylation levels are associated with clinically relevant cervical lesions and a high cervical cancer progression risk.

*FAM19A4/miR124‐2* methylation analysis was evaluated in cervical scrapes from a South African screening cohort of women living with HIV, showing good sensitivity and specificity for the detection of CIN3+ when used as a triage test of hrHPV‐positive women (sensitivity 72.9%, specificity 76.1%).^91^ As women living with HIV have a high CIN3+ risk, direct referral of HP16/18‐positive women and triage of only those with a non‐16/18 hrHPV infection with *FAM19A4/miR124‐2* methylation analysis is an interesting option to limit the number of tests performed while sensitivity and specificity are maintained (79.7% and 74.8%, respectively). The marker panel *CADM1/MAL/miR124‐2* was evaluated in this same cohort and a Kenyan cohort of women living with HIV, showing similar clinical performance when used as a triage marker (sensitivity 73.8% and 72%, specificity 81.5% and 70%, respectively).^88^, ^89^ *EPB41L3* was evaluated in cervical scrapes of women living with HIV from South Africa and Burkina Faso who were included in a prospective cervical screening study, showing a specificity of 66.2% at the threshold corresponding to 69.7% sensitivity for CIN2+, independent of hrHPV status.^90^

## Future directions

Currently available data justify further validation of methylation triage testing on cervical scrapes and self‐collected cervico‐vaginal specimens of hrHPV‐positive women in prospective implementation studies within cervical screening programmes in both high‐ and low‐resource settings, including a health‐economic evaluation of costs and benefits. Once similar data are available for methylation triage on self‐collected material and, potentially, urine, full molecular screening without the need for a visit to a clinician can be envisioned.^28^, ^61^ Given its high sensitivity for invasive cervical cancer and advanced CIN lesions, methylation analysis may eventually be considered as a primary cervical screening tool, either with or without hrHPV testing. Currently, methylation assays can be used following a positive HPV test, but are not yet available as point‐of‐care tests. However, the development of high‐throughput variants will change this. Future development of methylation assays into point‐of‐care tests would be ideal for low‐resource settings in order to facilitate same‐day screening and treatment and minimise loss to follow up.

### Disclosure of interests

RDMS, DAMH and CJLMM are minority shareholders of Self‐screen B.V., a spin‐off company of VUmc, which develops, manufactures and licences the high‐risk HPV assay and methylation marker assays for cervical cancer screening and holds patents on these tests. CJLMM is part‐time CEO of Self‐Screen, has stock in Qiagen and MDXHealth, has received speakers fees from GSK, Qiagen and SPMSD/Merck, and served occasionally on the scientific advisory boards (expert meeting) of these companies. DAMH has been on the speakers bureau of Qiagen and serves occasionally on the scientific advisory boards of Pfizer and Bristol‐Myers Squibb. WWK and GGK declare no conflicts of interests. Completed disclosure of interests forms are available to view online as supporting information.

### Contribution to authorship

WWK was responsible for the literature search and data acquisition. Data interpretation and writing of initial draft was by WWK and CJLMM. Manuscript writing and approval was by WWK, RDMS, DAMH, GGK and CJLMM.

### Details of ethics approval

Not applicable.

### Funding

CJLMM was in part supported by the European Research Council (ERC Advanced Masscare 322986).

## Supporting information


**Table S1.** Overview of individual and combinations of host cell methylation markers included in commercial or research assays and their performance for the detection of CIN3 or worse in cervical scrapes or self‐collected specimens in different clinical settings.

Supplementary Material

Supplementary Material

Supplementary Material

Supplementary Material

Supplementary Material
