# Supplementary material for: The use of host cell DNA methylation analysis in the detection and management of women with advanced cervical intraepithelial neoplasia: a review
Source: BJOG. 2020 Aug 9;128(3):504–14. doi: 10.1111/1471-0528.16395 (PMC7818489; doi:10.1111/1471-0528.16395)
Supplement: Supplementary file 1 — Table S1. Overview of individual and combinations of host cell methylation markers included in commercial or research assays and their performance for the detection of CIN3 or worse in cervical scrapes or self‐collected specimens in different clinical settings. [file BJO-128-504-s001.pdf]

Table S1. Overview of individual and combinations of host cell methylation markers included in commercial or research assays and their performance for the detection of CIN3 or worse in cervical scrapes or self-collected specimens in different clinical settings

Marker (panel)	Assay	Reference	HPV	Sample type	N	CIN3 (%)	ICC (%)	Sens.	Spec.	NPV	PPV	Comment
Screening populations												
FAM19A4	QMSP	[44]	HPV+	Cervical scrapes	218	30 (13.8%)	3 (1.4%)	75.8	67	93.9	29.1	Consecutive series from screening cohort
FAM19A4	QMSP	[62]	HPV+	Cervical scrapes	115	42 (36.5%)	2 (1.7%)	68.18	60.6	75.4	51.7	Endpoint CIN2+ (n=44); fixed number of women with normal cytology were included
CADM1	QMSP	[62]	HPV+	Cervical scrapes	115	42 (36.5%)	2 (1.7%)	70.5	60.6	76.8	52.5	Endpoint CIN2+ (n=44); fixed number of women with normal cytology were included
MAL	QMSP	[62]	HPV+	Cervical scrapes	115	42 (36.5%)	2 (1.7%)	72.7	50.7	75	47.8	Endpoint CIN2+ (n=44); fixed number of women with normal cytology were included
Non-attendee population												
MAL/miR124-2	QMSP	[31]	HPV+	Selfsamples	355	65 (18.3%)	5 (1.4%)	64.9-71.6	70	-	-	
MAL/miR124-2	QMSP	[32]	HPV+	Cervical scrapes and selfsamples	1019	134 (13.2%)	13 (1.3%)	68	55.3	91.1	20.4	Pooled performance on both sample types (505 scrapes, 514 selfsamples)
EPB41L3	QMSP	[36]	HPV+	Cervical scrapes<br>Selfsamples (n=49)	128	25 (19.5%)	9 (7.0%)	88<br>79	61<br>88	-<br>-	-<br>-	Matching scrapes and selfsamples; fixed number of women with normal cytology were included
Gynaecological referral populations/Selection of archived material												
FAM19A4	QMSP	[61]	HPV+	Selfsamples	450	73 (16.2%)	2 (0.4%)	65.3	81.3	92.1	41.2	Gynaecological referral population
FAM19A4	QMSP	[60]	HPV+	Cervical scrapes	508	87 (17.1%)	3 (0.6%)	75.6	71.1	93.1	36	Gynaecological referral population
FAM19A4	QMSP	[29]	HPV+	Cervical scrapes	215	57 (26.5%)	61 (28.4%)	75.4	81.4	-	-	Gynaecological referral population; endpoint HSIL+ (n=57)
miR124-2	QMSP	[63]	HPV+/-	Cervical scrapes	407	128 (31.4%)	-	86.7	61.3	-	-	Colposcopy referral population; endpoint CIN2+ (n=128)
MAL	PSQ	[65]	HPV+	Cervical scrapes	884	208 (23.5%)	9 (1.1%)	94	6	-	-	Women with abnormal cytology referred for colposcopy; endpoint CIN2/3 (n=330)
EPB41L3/HPV16L1/HPV16L2/HPV18L2/HPV31L1[a]	PSQ	[69]	HPV+	Cervical scrapes	1493	373 (25%)	-	90	36	-	-	Women with abnormal cytology referred for colposcopy; endpoint CIN2/3 (n=556)
EPB41L3	QMSP	[63]	HPV+/-	Cervical scrapes	407	128 (31.4%)	-	63.3[b]	84.2[b]	-	-	Colposcopy referral population; endpoint CIN2+
EPB41L3	QMSP	[35]	HPV+	Cervical scrapes	152	59 (38.8%)	43 (28.3%)	85	68	-	-	Gynaecological referral population
EPB41L3	QMSP	[68]	HPV+/-<br>HPV+	Cervical scrapes	143<br>101	37 (25.9%)<br>35 (34.7%)	2 (1.4%)<br>2 (2.0%)	67[b]<br>70	85[b]<br>83	-	-	Women with abnormal cytology
EPB41L3	PSQ	[65]	HPV+	Cervical scrapes	884	208 (23.5%)	9 (1.1%)	85	31	-	-	Women with abnormal cytology referred for colposcopy; endpoint CIN2/3 (n=330)
DLX1/ITG4/RXFP3/SOX17/ZNF671	QMSP	[38]	HPV+	Cervical scrapes	217	25 (11.5%)	19 (8.8%)	76.2	82.9	-	-	Colposcopy referral population
PAX1	PSQ	[46]	HPV+/-	Cervical scrapes	121	20 (16.5%)	27 (22.3%)	44.1[b]	95[b]	-	-	Endpoint CIN2/3 (n=34)

a Combination of cellular and viral targets
b Performance evaluated independent of HPV result

Abbreviations: CIN, cervical intraepithelial neoplasia, graded 1–3; HPV, human papillomavirus; HSIL, high-grade squamous intraepithelial lesion; ICC, invasive cervical cancer; N, sample size; NPV, negative predictive value; PPV positive predictive value; PSQ, pyrosequencing; QMSP; quantitative methylation-specific polymerase chain reaction; Sens., sensitivity; Spec., specificity.
